# Single-cell Protein and Xylitol Production by a Novel Yeast Strain *Candida intermedia* FL023 from Lignocellulosic Hydrolysates and Xylose

**DOI:** 10.1007/s12010-017-2644-8

**Published:** 2017-11-02

**Authors:** Jiaqiang Wu, Jinlong Hu, Shumiao Zhao, Mingxiong He, Guoquan Hu, Xiangyang Ge, Nan Peng

**Affiliations:** 1 State Key Laboratory of Agricultural Microbiology, College of Life Science and Technology, Huazhong Agricultural University, Wuhan, 430070 People’s Republic of China; 2 Key Laboratory of Development and Application of Rural Renewable Energy (Ministry of Agriculture), Biomass Energy Technology Research Centre, Biogas Institute of Ministry of Agriculture, Chengdu, 610041 Sichuan People’s Republic of China

**Keywords:** *Candida intermedia*, Single-cell protein, Xylitol, Lignocellulosic hydrolysate, Simultaneous saccharification and fermentation

## Abstract

Yeasts are good candidates to utilize the hydrolysates of lignocellulose, the most abundant bioresource, for bioproducts. This study aimed to evaluate the efficiencies of single-cell protein (SCP) and xylitol production by a novel yeast strain, *Candida intermedia* FL023, from lignocellulosic hydrolysates and xylose. This strain efficiently assimilated hexose, pentose, and cellubiose for cell mass production with the crude protein content of 484.2 g kg^−1^ dry cell mass. SCP was produced by strain FL023 using corncob hydrolysate and urea as the carbon and nitrogen sources with the dry cell mass productivity 0.86 g L^−1^ h^−1^ and the yield of 0.40 g g^−1^ sugar. SCP was also produced using NaOH-pretreated *Miscanthus sinensis* straw and corn steep liquor as the carbon and nitrogen sources through simultaneous saccharification and fermentation with the dry cell productivity of 0.23 g L^−1^ h^−1^ and yield of 0.17 g g^−1^ straw. *C. intermedia* FL023 was tolerant to 0.5 g L^−1^ furfural, acetic acid, and syringaldehyde in xylitol fermentation and produced 45.7 g L^−1^ xylitol from xylose with the productivity of 0.38 g L^−1^ h^−1^ and the yield of 0.57 g g^−1^ xylose. This study provides feasible methods for feed and food additive production from the abundant lignocellulosic bioresources.

## Introduction

There is a growing interest in utilization of renewable bioresources for production of biochemicals (such as xylitol and lactic acid) and single-cell proteins (SCPs). Renewable bioresource provides cost-effective feedstock as it is the most abundant source of sugars and does not compete with food resources [29]. Potential renewable bioresource substrates include corncobs and stalks, sugarcane press mud, and *Miscanthus* straw; however, the deconstruction of cellulosic polymers through hydrolysis and saccharification requires additional processing that increases the cost and difficulty of lignocellulose-to-product conversion [18]. Xylose is the second most abundant sugar in lignocellulose after glucose [26]; therefore, efficient and rapid utilization of xylose is a prerequisite for biochemicals and SCP production.

The production of sufficient protein from livestock in Asia, particularly China, represents a serious challenge for the future. Food-producing animals rely heavily on soybean meal, fishmeal, and corn [3]. Increasing prices for the most important agricultural crops will lead to an increase in prices for beef, pork, and poultry [36]. Food scarcity may become even more serious in light of the rising demand for biofuels and subsequent reductions in agricultural productivity. Land and labor productivity grew at a substantially slower rate from 1990 to 2005 than from 1961 to 1990 [1]. SCP is a major protein substitute and its bioconversion from agricultural residues and industrial wastes makes it cheaper than traditional protein sources [2]. Algae, fungi, and bacteria are the chief sources of microbial protein that can be used as SCP. Many fungal species are used as protein-rich food, including the most common yeasts *Candida*, *Hansenula*, *Pichia*, *Torulopsis*, and *Saccharomyces*. Many other filamentous fungi also serve as sources of SCP [2]. *Chaetomium cellulolyticum*, a cellulolytic fungus, degrades lignocellulose via a secreted cellulase to produce SCP [10, 24, 25]. *Chaetomium* strains, however, with the exception of *C. cellulolyticum*, produce mycotoxins that are cytotoxic in HeLa cells [34]. *Aspergillus terreus* degrades alkali-pretreated sugarcane bagasse to produce SCP; optimum conditions yield a protein content of 210–280 g kg^−1^ [7]. *Candida tropicalis*, *C. langeronii*, and *C. arborea* have been used with sugar cane bagasse and rice straw hydrolysate to produce SCP [27, 30, 42]. Productivities were 0.2 and 0.97 g L^−1^ h^−1^ and yields were 0.31 and 0.40 g g^−1^ for *C. tropicalis* and *C. langeronii*, respectively. *Debaryomyces hansenii*, *Kluyveromyces marxianus*, and *Pichia stipitis* assimilate non-detoxified hemicellulosic hydrolysate from spent brewery grains; *D. hansenii* showed the best performance with a productivity of 0.56 g L^−1^ h^−1^ [6].

Xylitol is categorized as a polyalcohol or sugar alcohol with a wide range of uses in medical and food industries. However, the traditional production method involves the reduction of d-xylose to xylitol via a catalytic chemical reaction with high pressure and temperature resulting in a complicated process, equipment, and large investment [23]. Biotechnological xylitol production is a potentially attractive replacement for the chemical process, as it occurs under much milder process conditions and can be based on sugar mixtures such as lignocellulosic hydrolysates to save on energy and substrate purification costs [15]. Biotechnological conversion is mostly accomplished by whole cell biocatalyst, involving bacteria, fungi, yeast and/or recombinant strains, or cell-free/immobilized enzyme system. Several challenges emerged in xylitol fermentation from lignocellulosic materials, most of them related to hydrolysis of biomass, detoxification, fermentation process factors, etc. For example, inhibitors, such as furfural, acetic acid, and syringaldehyde, generated from acid or alkaline pretreatments of lignocellulosic materials are toxic to microorganisms and inhibitory to cellulase, resulting in low fermentation efficiency [9, 22]. Therefore, isolating potential species with tolerance to inhibitors is important for xylitol production from lignocellulosic materials.

Although efforts are being made to improve SCP and xylitol production from agricultural lignocellulose residues, an efficient and effective method has yet to be developed. In this study, we isolated a novel *C. intermedia* strain FL023 which was tolerant to furfural, acetic acid, and syringaldehyde, mimicking the main inhibitor from alkaline-pretreated lignocellulosic materials. *C. intermedia* FL023 utilized lignocellulosic hydrolysate for high-efficiency SCP production and utilized xylose for xylitol production. Taken together, this study provided potential methods for SCP and xylitol production from renewable bioresources.

## Materials and Methods

### Screening for High-Efficiency Xylose-Utilization Yeast

Six yeast strains were obtained from the Microbial Genetic Stock Centre (MGSC) of State Key Laboratory of Agricultural Microbiology, Wuhan, China. All strains were maintained on yeast extract peptone dextrose (YPD) agar tube slants (glucose 20 g L^−1^, peptone 20 g L^−1^, yeast extract 10 g L^−1^, and agar 20 g L^−1^). For screening, yeast strains were inoculated in YPD medium and cultured at 30 °C for 48 h with shaking. An aliquot (0.5 mL) of preculture was transferred into 50 mL yeast extract peptone xylose (YPX) medium (xylose 20 g L^−1^, peptone 5 g L^−1^, yeast extract 3 g L^−1^) and incubated at 30 °C for 48 h with shaking. After incubation, viable cell counts were determined by standard plate counting, dry cell weight was measured, and cell mass yields were calculated. The yeast strain with the highest xylose-dry cell weight conversion efficiency was selected for further study. The growth curve of *C. intermedia* cells in YPD, YPX, YPC (20 g L^−1^ cellobiose instead of glucose in YPD medium), and YPDX (6.67 g L^−1^ glucose and 13.33 g L^−1^ xylose instead of the sugar in YPD medium) was generated at 30 °C for 24 h with shaking at 250 rpm. During fermentation, the optical density at 600 nm (OD_600_) was measured by spectrophotometry every 2 h.

### Measurement of Reducing Sugar

The reducing sugar was assayed by the 3,5-dinitrosalicylic acid colorimetric method (DNS method) [12]. Briefly, 1-mL sample was mixed with 3-mL DNS reagent in the colorimetric tube. After boiling for 5 min in a water bath, the volume was brought to 25 mL and absorbance was measured at OD_540_. A standard curve of glucose or xylose was used to determine the reducing sugar concentration. Reducing sugar released from straw and xylitol were determined by HPLC (Agilent 1200) on an Agilent Zorbax Carbohydrate Analysis Column (4.6 × 250 mm, 5 μm). All tests were done in triplicate.

### Taxonomic Identification and Phylogenetic Analysis

The selected yeast strain was identified by determining the sequences of the D1/D2 domain of the 26S rDNA. The partial 26S rDNA sequence was PCR amplified using primers NL-1 and NL-4 [16]. The PCR product was separated by agarose gel electrophoresis, then purified with a PCR Clean-up Kit (Axygen, Hangzhou, China) and sequenced at Invitrogen (Shanghai, China). The sequence was compared against the GenBank database (www.ncbi.nlm.nih.gov) and the new 26S rDNA sequence was deposited under accession number KF792071. The sequence of the 26S rDNA D1/D2 domain was aligned with the sequences of xylose-fermenting yeast strains retrieved from NCBI and the resulting tree was plotted using MEGA5 [35].

### Morphological and Physiological Characterization

The macroscopic morphology of yeast grown on YPD or cornmeal agar plate for 1 or 5 days was observed under a general microscope. Growth at different temperatures (30, 37, 42, and 45 °C) was performed in YPX medium for 48 h with shaking and measured as OD_600_. Sugar utilization tests were carried out using basal medium (5 g L^−1^ peptone and 3 g L^−1^ yeast extract, pH 5) plus 20 g L^−1^ sugar (including d-galactose, maltose, sucrose, d-cellobiose, d-raffinose, lactose, L-arabinose, d-glucose, d-mannose, d-trehalose, and d-xylose). Cells were cultured in a flask at 30 °C for 48 h with shaking. Total crude protein content was estimated from the total nitrogen content by the Kjeldahl method [19].

### Optimization of Nitrogen Source for Cell Growth

Nitrogen source selection was optimized by fractional factorial design as described previously [20]. NH_4_NO_3_, NH_4_Cl, (NH_4_)_2_SO_4_, (NH_4_)_2_HPO_4_, and urea were added to XYNB medium (30 g L^−1^ xylose, 3.4 g L^−1^ YNB). The C:N ratio was set to 12:1 by bringing each nitrogen source to a concentration of 1.42, 1.91, 2.36, 2.36, and 1.07 g L^−1^, respectively. The fractional factorial design is shown in Table 4. Cells were inoculated in flasks and shaken at 250 rpm and 30 °C for 48 h. Dry cell weight and yields were calculated in triplicate experiments. Experimental design and statistical analyses were performed in Minitab statistical software (Minitab17).

### Continuous Feeding Fermentation for Yeast Cells Using Corncob Acid Hydrolysate

The corncob was dried at 60 °C to a constant weight and then hydrolysed at 121 °C for 90 min using 1% sulphuric acid with a liquid/solid ratio of 8. The hydrolysate was filtered and adjusted to pH 5.0. The inoculum seed culture of *C. intermedia* strain FL023 was grown in YPD medium with constant shaking (250 rpm) at 30 °C until an OD_600_ of 20 was reached. Seed culture (0.3 L) was added to a 10-L automatic continuous-feeding fermenter (DRS71S6, SEW Eurodrive, USA) containing 3 L of the corncob hydrolysate medium (20 g L^−1^ reducing sugar, 3.4 g L^−1^ YNB, and 2 g L^−1^ KH_2_PO_4_). The reactor was agitated at 250 rpm and aeration was maintained at 2 vvm. After fermentation for 12 h, fresh concentrated hydrolyte feed medium (160 g L^−1^ reducing sugar, 2 g L^−1^ KH_2_PO_4_, and 20 g L^−1^ urea) was added at 0.143 L h^−1^ for 28 h (4 L total). During feeding, the reaction was agitated at 300 rpm, and aeration maintained at 3.0 vvm. Throughout fermentation, the pH was maintained at 5.0 by automatic feeding of ammonia. Reducing sugar concentration and dry cell weight per liter culture were determined during fermentation.

### Simultaneous Saccharification and Fermentation Production of Yeast Cells with *Miscanthus* Straw Substrate

*C. intermedia* strain FL023 was grown at 30 °C in YPX medium. At OD_600_ = 20, cells were used for inoculation. *Miscanthus* straw (50 g L^−1^, 100 mesh) pretreated with 1.25 M NaOH and each nitrogen source [10 g L^−1^ yeast extract; or 10 g L^−1^ corn steep liquor (CSL), 0.64 g L^−1^, urea and 1.41 g L^−1^ (NH_4_)_2_SO_4_ corresponding to the same amounts of nitrogen of 10 g L^−1^ yeast extract] was mixed with H_2_O up to 40 mL in a 500-mL flask, adjusted to pH 5.0, and autoclaved at 115 °C for 20 min. After cooling the autoclaved suspension of cellulosic material, 10% (*v*/*v*, 5 mL) yeast starter culture (1 × 10^9^ mL^−1^) and 10 filter paper units (FPU) per gram substrate of cellulase (Celic2, Novozymes, Denmark) were added up to 50 mL medium for simultaneous saccharification and fermentation (SSF). The cell culture was incubated at 30 °C with shaking for 72 h. Cell numbers were quantified by viable counting and reducing sugars were measured by HPLC.

### Shaking-Flask Fermentation for Xylitol

*C. intermedia* strain FL023 was pregrown overnight in rich nutrition medium containing 20 g L^−1^ xylose, 20 g L^−1^ peptone, and 10 g L^−1^ yeast extract. After that, cells were collected, washed twice with water, and resuspended in xylose medium (5 g L^−1^ peptone, 3 g L^−1^ yeast extract and xylose) for fermentation. The fermentation process was conducted through two stages: aerobic cell growth and microaerobic xylitol production. For aerobic growth, the strain was inoculated for 12 h at 30 °C with a starting OD_600_ of 1.0 at 240 rpm; for microaerobic xylitol production, fermentation was carried out for 72 h at 140 rpm and 30 °C. To test the inhibitor tolerance, cells were cultured in xylose medium with 0.5 g L^−1^ furfural, acetic acid, or syringaldehyde, respectively. One-milliliter samples were removed at the indicated time points to test the growth of the culture by spectrophotometer and 50 μL supernatants were analyzed to determine xylose and xylitol concentrations by HPLC.

### Production of Xylitol from Xylose in a 5-L Bioreactor

15% (*v*/*v*) seed culture of *C. intermedia* strain FL023 (300 mL) pregrown overnight in rich nutrition medium containing 20 g L^−1^ xylose, 20 g L^−1^ peptone, and 10 g L^−1^ yeast extract was inoculated in the fermentation medium containing 80 g L^−1^ xylose and 10 g L^−1^ corn steep liquor (CSL) in 2 L working volume. Aerobic cell growth at 30 °C for 24 h at 240 rpm with aeration of 0.6 vvm and sequential microaerobic xylitol production at 30 °C for 96 h at 140 rpm with aeration of 0.4 vvm were conducted in this study. To test the rate of production of xylitol and the growth of cells, 2-mL samples were removed at the indicated time points. The culture was tested by spectrophotometer at OD 600 nm and 50 μL supernatant was dissolved in dilute sulphuric acid to analyze xylose and xylitol concentrations by HPLC.

### Statistical Analysis

All experiments were conducted in triplicate. Data were analyzed using Excel and the mean ± SD were calculated. Experimental design and statistical analyses for optimization of nitrogen sources were performed in Minitab statistical software (Minitab17).

## Results

### Isolation of a High-Efficiency Xylose-Utilization Yeast Strain

**Fig. 1:**
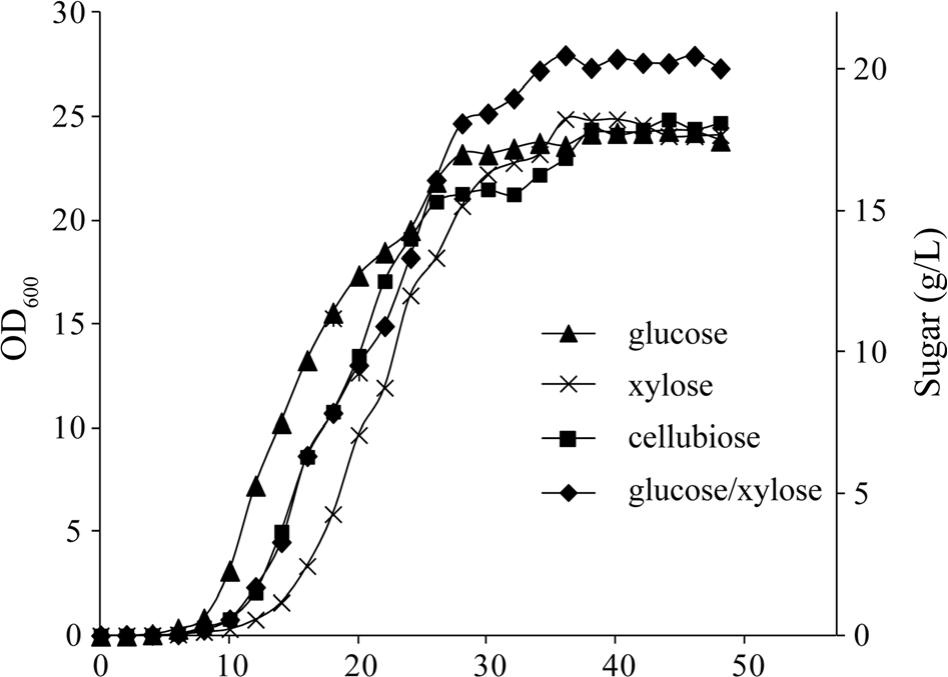
Growth curve of *C. intermedia* FL023 in 20 g L^−1^ glucose, xylose, cellobiose, and mixed sugar (6.67 g L^−1^ glucose and 13.33 g L^−1^ xylose) media

Cellulosic hydrolysates consist of 600–700 g kg^−1^ glucose and 300–400 g kg^−1^ xylose [26]. However, inefficient xylose usage is the main obstacle to exploring lignocellulose resources. Six previously isolated yeast strains were screened for high-efficiency xylose utilization. All strains were cultured in YPX medium at 30 °C and 250 rpm for 48 h. Under these conditions, ethanol fermentation was inhibited and most of the sugar was used for cell growth. As shown in Table 1, cell number, dry cell weight, and yields of strain FL023 were the highest; Y28 and Y7 had the lowest yields. Thus, as judged by all three parameters, the strain FL023 was better than the other candidate strains and was selected for further study. Growth of FL023 was assessed in glucose, xylose, cellobiose, and glucose-xylose media. FL023 grew quickly in all tested media; the OD_600_ reached 25 at stationary phase (Fig. 1). It showed average productivities of 0.21, 0.22, 0.21, and 0.25 g L^−1^ h^−1^, and yields of 0.38, 0.39, 0.40, and 0.44 g g^−1^ in glucose, xylose, cellobiose, and mixed-sugar media. FL023 grew faster in glucose medium during exponential phase and had a longer lag phase in xylose medium than that in glucose medium. Xylose, one of the major sugar in lignocellulose hydrolysates, was quickly consumed at the first 25 h. Importantly, cellobiose, one of the main factors in feedback inhibition of cellulose hydrolysis, was quickly assimilated by FL023 (Fig. 1). All these results suggested the strain FL023 has great potential in SCP production using the lignocellulose as the carbon source.

**Table 1:** Screening for efficient pentose-assimilation yeast strain

Strains	Viable count (×10^9^ cfu mL^−1^)	Cell dry weight (g L^−1^)	Yield (g g^−1^)
FL023	1.26	8.1	0.40
FL621	0.44	3.1	0.16
Y3	0.51	3.4	0.17
Y7	0.52	3.3	0.17
Y10	0.58	5.4	0.27
Y28	0.66	4.4	0.22

### Taxonomic Identification and Phylogenetic Analysis

**Fig. 2:**
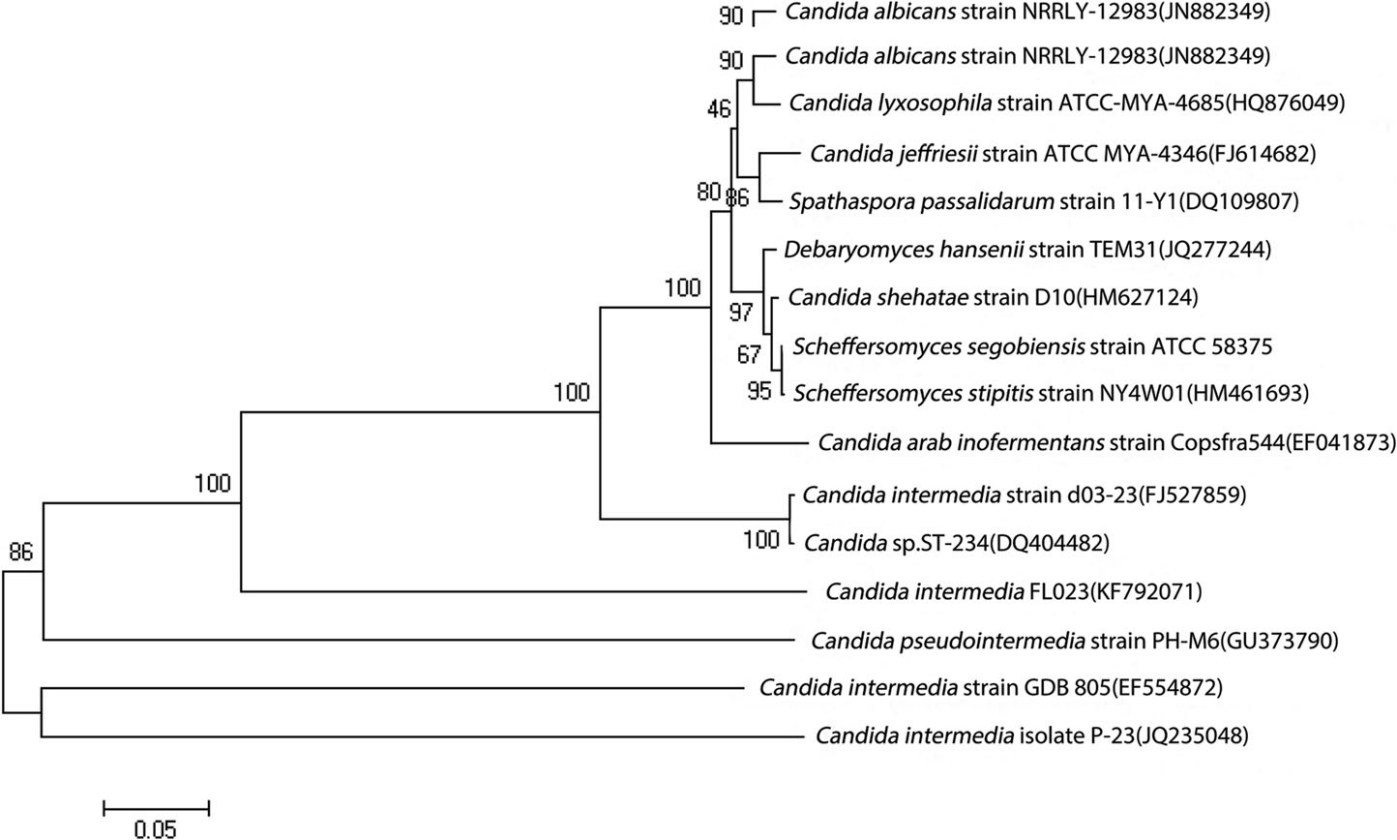
Phylogenetic tree generated by neighbor-joining analysis based on the D1/D2 region of the 26S rDNA sequence of *C. intermedia* FL023 and pentose-assimilating yeast. Numbers on the branches represent bootstrap values; accession numbers for the 26S rDNA sequences follow the strain names

The sequence of the D1/D2 domain of the 26S rDNA from the strain FL023 (NCBI accession number, KF792071) was amplified and searched against the NCBI database. The sequence was most similar to *Candida intermedia* strain VVT C-04520 (NCBI accession, DQ377635) [17]. Thus, the strain was named *C. intermedia* FL023. Differences between strain FL023 and strain VVT C-04520 included six substitutions in the 558 nucleotides (98.9% identity) in the D1/D2 domain of the 26S rDNA sequence. According to previous studies, *Candida* strains with > 1% nucleotide substitutions in the D1/D2 domain likely represent different species [16]. Thus, strain FL023 represents a novel *C. intermedia* species. The phylogenetic tree of 26S rDNA D1/D2 sequences is shown in Fig. 2. *C. intermedia* FL023 formed a close lineage with all *Candida* species except *C. intermedia* strain GDB 805, *C. intermedia* isolate P-23, and *C. pseudointermedia* strain PH-M6 (bootstrap value 86%).

### Morphological and Physiological Characterization

The physiological characteristics of *C. intermedia* FL023 are summarized in Table 2. The optimal pH for growth of strain FL023 was 5.0 in YPD medium; the strain grew at pH 4.4–7.6, formed a pulvinate, smooth, ivory-white colony on YPD agar, and did not become dry or wrinkled. The strain grew well at 28 and 30 °C and grew weakly at 37 °C in YPD and YPX media. It assimilated all tested sugars well, except d-galactose, α-lactose, and L-arabinose. FL023 was not able to produce ethanol from xylose. Microscopic observation of FL023 grown on YPD medium revealed that the strain was ellipsoidal in shape, ranging from 3.5–5.5 μM at large diameter and 2.9–4.5 μM at small diameter, proliferated by budding, and produced oval cells in a chain of elongated cells. However, pseudohyphae were not observed on YPD or cornmeal agar plates. Strain FL023 showed higher tolerance against NaCl (Table 2). This strain contained 48.4% crude proteins based on total nitrogen measurement. The dry cell weight of strain FL023 sample is correlated to either the OD_600_ or the number of cells per volume. It was estimated that OD_600_ = 1 was equivalent to 0.31 g L^−1^, and 10^9^ cells mL^−1^ was equivalent to 0.65 g of dried cells per liter. It could therefore be used to estimate cell mass production during SSF.

**Table 2:** Phenotypic characteristics of *Candida intermedia* FL023

Spore formation	–				
Pseudohyphae formation	–				
Ethanol fermentation	w				
Assimilation					
d-glucose	+++	d-xylose	+++	d-galactose	++
L-arabinose	+	Sucrose	+++	d-trehalose	++
maltose	+++	α-lactose	++	d-cellobiose	+++
d-raffinose	+++				
Growth temperature					
28 °C	+++	30 °C	+++		
37 °C	w	40 °C	–		
NaCl tolerance					
12%	+++	14%	+++		
16%	w	18%	w		

Positive +++, ++, +; − negative; w weak

### Inorganic Nitrogen Source Optimization

A fractional factorial design was used to find the best nitrogen resource for optimal xylose conversion. Five low-cost nitrogen resources [urea, NH_4_NO_3_, NH_4_Cl, (NH_4_)_2_SO_4_, and (NH_4_)_2_HPO_4_] were used in this experiment. The medium consisted of 20 g L^−1^ xylose and each nitrogen source (the mass ratio of C:N is 12:1). The fractional factorial design and results are shown in Table 3. The significance analysis was performed in Minitab statistical software (Minitab17; Table 4). Urea significantly improved xylose conversion (*p* = 0.019). (NH_4_)_2_SO_4_ had a slightly positive effect (Table 4). Other nitrogen sources had a negative effect on xylose conversion; thus, urea was selected as the nitrogen source for fermentation of corncob hydrolysate and *Miscanthus* straw.

**Table 3:** Fractional factorial design for optimal nitrogen source for *C. intermedia* growth

No.	Urea	NH_4_NO_3_	NH_4_Cl	(NH_4_)_2_SO_4_	(NH_4_)_2_HPO4	Dry cell weight (g L^−1^)	Yield
1	1.071	0.000	0.000	0.000	0.000	7.00	0.2345 ± 0.02
2	0.000	0.000	1.911	2.357	0.000	4.00	0.1741 ± 0.04
3	1.071	1.42	0.000	2.357	0.000	6.50	0.2223 ± 0.03
4	0.000	0.000	0.000	2.357	2.357	4.5	0.1749 ± 0.03
5	1.071	0.000	1.911	0.000	2.357	5.75	0.1951 ± 0.01
6	1.071	1.420	1.911	2.357	2.357	5.5	0.1860 ± 0.05
7	0.000	1.420	1.911	0.000	0.000	4.25	0.1443 ± 0.03
8	0.000	1.420	0.000	0.000	2.357	5.00	0.1696 ± 0.01

**Table 4:** Significant analysis of optimal nitrogen sources for production of yeast cells

Argument	Coefficient	Standard error of regression coefficient	*T*	*p*
Constant	0.189975	0.007561	25.12	0.002
Urea	0.040850	0.005765	7.09	0.019*
NH4NO_3_	− 0.009930	0.004348	− 2.28	0.150
NH4Cl	− 0.013318	0.003231	− 4.12	0.054
(NH4)_2_SO_4_	0.001464	0.002619	0.56	0.633
(NH4)_2_HPO_3_	− 0.005261	0.002619	− 2.01	0.182

*p value < 0.05 means an efficient improvement to the yield

### Efficient Transformation of Corncob Hydrolysate into Yeast Cells

**Fig. 3:**
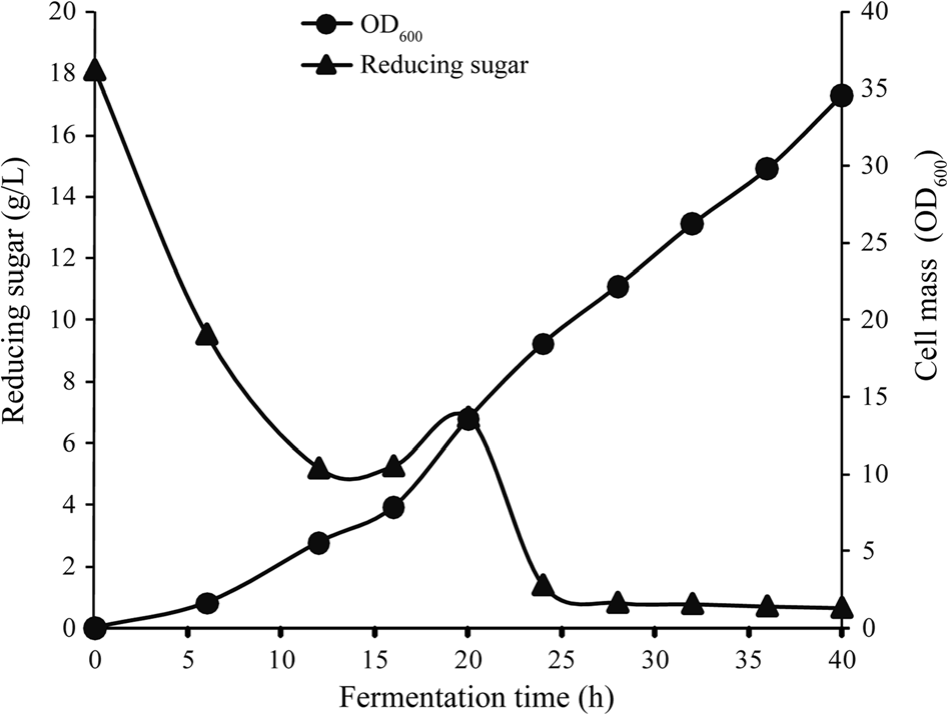
*C. intermedia* FL023 growth in corncob acid hydrolysate by continuous fed-batch fermentation. Nitrogen source, 20 g L^−1^ urea. Continuous feeding began at 12 h

Fermentation was performed at 30 °C for 40 h with aeration at 2.0 vvm with corncob acid hydrolysate and urea as the carbon and nitrogen sources. The starter culture contained 3-L medium (6.71 g L^−1^ glucose and 13.29 g L^−1^ xylose), and the continuous feeding medium contained 4 L concentrated corncob acid hydrolysate (160 g L^−1^ reducing sugar) and 20 g L^−1^ urea (Fig. 3). This method yielded average sugar and urea concentrations of 86.14 and 11.32 g L^−1^. Reducing sugar was assimilated quickly; at 12 h, continuous feeding with sugar was initiated. The sugar concentration slightly increased after feeding until 19 h, after which the concentration dropped quickly. After 25 h, the sugar was nearly depleted; cell mass increased throughout the process and finally reached OD_600_ of 35, which was equal to 34.6 g L^−1^ (Fig. 3). The productivity was 0.86 g L^−1^ h^−1^ and yield was 0.40 g g^−1^.

### Efficient Transformation of *Miscanthus* Straw into Yeast Cells

*Miscanthus* has been identified as a biomass crop with global potential [21]. However, lignocellulose has been designed by nature to resist degradation [8]. Steam pretreatment with dilute mineral acids is an efficient approach to depolymerizing hemicellulose (200–400 g kg^−1^ of biomass dry weight) into sugars (hemicellulose hydrolysate, primarily xylose) and to increase access of cellulase enzymes [8]. However, side reaction products (furfural, 5-hydroxymethylfurfural, formate, acetate, and soluble lignin products) formed during pretreatment hinder fermentation [22]. In contrast, SSF enables fermentation of lignocellulose [28].

**Fig. 4:**
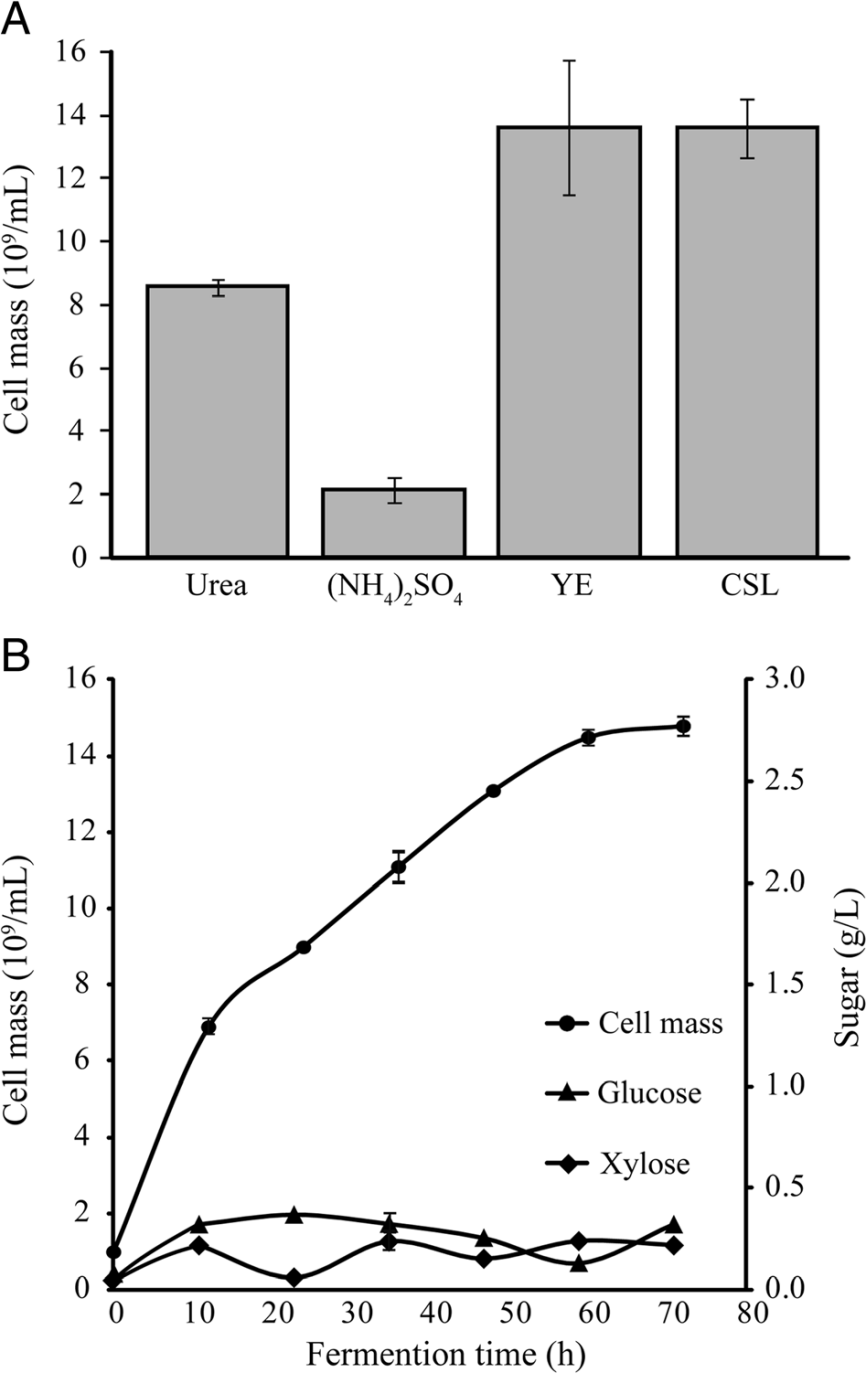
*C. intermedia* FL023 growth in 50 g L^−1^ NaOH-pretreated *Miscanthus* straw media under simultaneous saccharification and fermentation (SSF) conditions. Ten filter paper units Cellic2 per gram dry straw was added. **a** 0.64 g L^−1^ urea, 1.41 g L^−1^ (NH_4_)_2_SO_4_, 10 g L^−1^ yeast extract (YE), or 10 g L^−1^ corn steep liquor (CSL) served as the nitrogen source. **b** Cell mass accumulation and reducing sugar release during fermentation using CSL as the nitrogen source

The SSF process for SCP production from *Miscanthus* straw substrate was used in this study. Prior to SSF, the straw was crushed to 100-mesh size (average 150 μm) and pretreated with 1.5 M NaOH. SSF was performed at 30 °C for 72 h in fermentation medium containing 50 g L^−1^ pretreated *Miscanthus* straw, 10 g L^−1^ yeast extract [or 0.64 g L^−1^ urea, 1.41 g L^−1^ (NH_4_)_2_SO_4_, or 10 g L^−1^ CSL], and 10 FPU g^−1^ substrate cellulase (Cellic2) in a total volume of 100 mL. With yeast and CSL as the organic nitrogen resources, the cell masses were 13.6 × 10^9^ mL^−1^, equal to 8.4 g dry cell L^−1^ culture (Fig. 4a). Cell masses were 8.6 × 10^9^ mL^−1^ and 2.2 × 10^9^ mL^−1^, equal to 5.6 g and 2.1 g dry cell L^−1^ culture in media containing urea and (NH_4_)_2_SO_4_. Productivity was 0.23, 0.23, 0.16, and 0.06 g L^−1^ h^−1^ with yields of 0.17, 0.17, 0.11, and 0.04 g g^−1^ in media containing yeast extract, CSL, urea, and (NH_4_)_2_SO_4_, respectively. Although urea was the best nitrogen source in corncob hydrolysate fermentation, it was not suitable in SSF; the cost-effective alternative CSL worked well. Furthermore, cell mass accumulation and reducing sugar release were monitored during fermentation of *Miscanthus* straw and CSL (Fig. 4b). Throughout the process, sugar (glucose and xylose) released from *Miscanthus* straw remained low in broth, and cell mass accumulated quickly. Cell mass productivity was at its greatest in the first 10 h and slowed thereafter, at which time xylose assimilation might accelerate from 10 to 20 h. A similar relationship was observed between cell mass productivity and xylose assimilation throughout the process, suggesting catabolite repression was the main hindrance to productivity.

### Xylitol Production and Inhibitor Tolerance

**Fig. 5:**
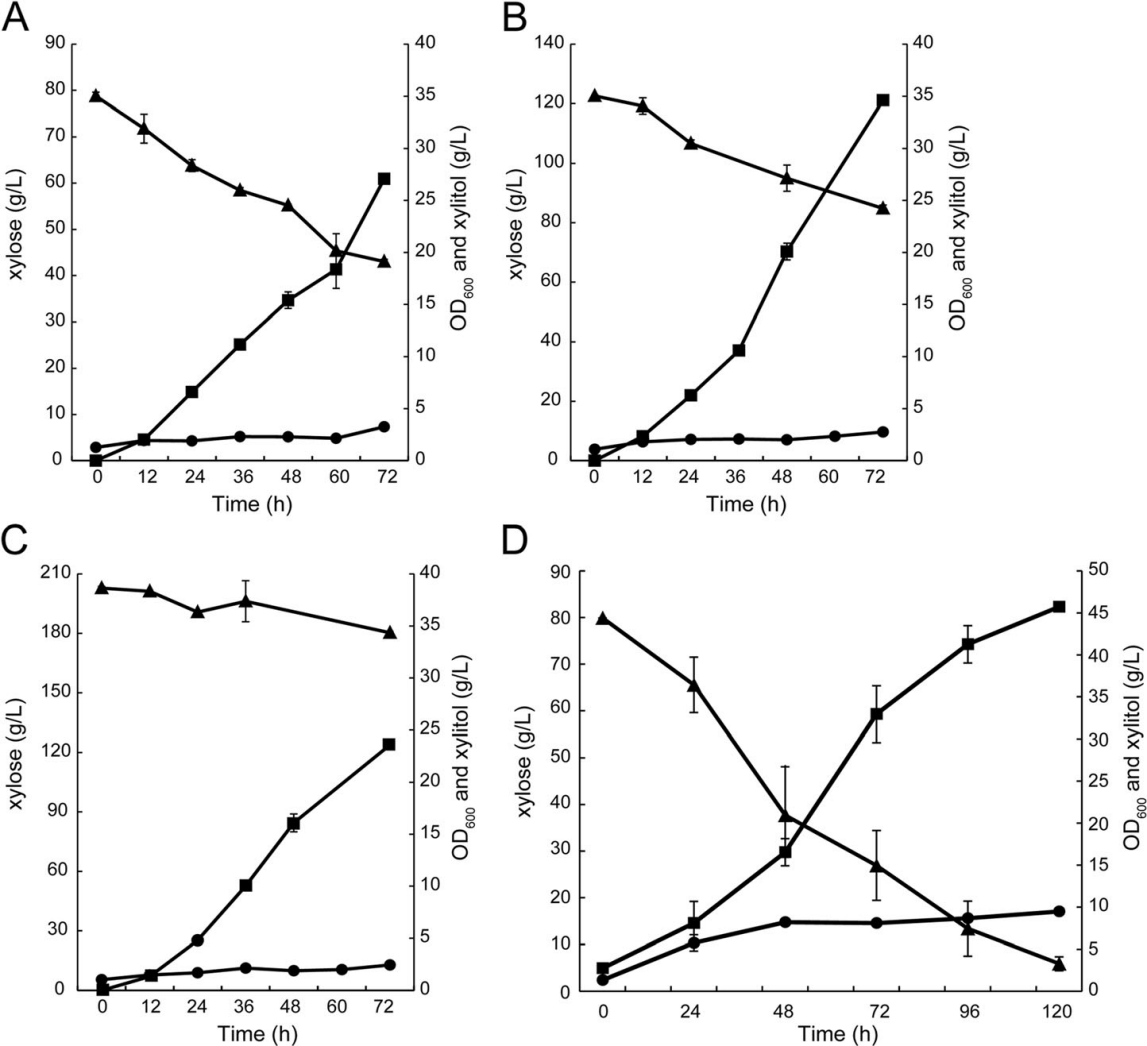
Xylitol fermentation from xylose in flasks and a 5-L bioreactor. Xylitol fermentation in flasks with **a** 80 g L^−1^, **b** 120, and **c** 200 g L^−1^ xylose at 30 °C; **d** xylitol fermentation in a 5-L bioreactor with 80 g L^−1^ xylose as substrate at 30 °C. Triangle, xylose; square, xylitol; and circle, cell density (OD_600_)

*C. intermedia* FL023 produces little ethanol from xylose and accumulates xylitol in the medium. At the flask level, 27.1 g L^−1^ xylitol was produced the medium containing 80 g L^−1^ xylose (Fig. 5a); while more xylitol (34.6 g L^−1^) was produced from the medium containing 120 g L^−1^ xylose (Fig. 5b). However, at these conditions, only 35.8 and 37.8 g L^−1^ xylose were consumed (Fig. 5a and b). These results indicated that xylose-to-xylitol transformation efficiency was high (0.76 and 0.92 g g^−1^); however, the xylitol productivities were very low. Increasing the xylose concentration to 200 g L^−1^ in the medium, however, reduced the xylitol titer to 23.6 g L^−1^, indicating high substrate concentration inhibited xylitol production ability significantly of *C. intermedia* FL023 (Fig. 5c). It is clear that the production rate and productivity of xylitol increased with the increase of initial xylose titer but the conversion efficiency would be reduced if the xylose concentration was too high. This is because xylose reductase (XR) activity is significantly affected by xylose concentration, which would ferment d-xylose into xylitol with NADPH as a cofactor; and excessive xylose increases the osmotic pressure, resulting in substrate inhibition [5, 37, 40]. Therefore, xylitol fermentation with extended time and low initial xylose substrate was conducted in a 5-L bioreactor under a microaerobic condition. At 72 h in the bioreactor, more xylose was consumed (residual xylose titer 43.0 vs 26.9 g L^−1^, Fig. 5d) and more xylitol was produced (27.1 vs 32.9 g L^−1^, Fig. 5d), indicating strict aeration condition facilitated xylitol fermentation. With extension of fermentation time, xylose was nearly completely consumed at 120 h with residual xylose of 5.9 g L^−1^. Finally, the xylitol titer, yield, and productivity reached 45.7 g L^−1^, 0.57 g g^−1^ xylose, and 0.38 g L^−1^ h^−1^. However, the xylose-to-xylitol transformation efficiency was low, and more cell mass was detected (Fig. 5d), indicating a part of xylose was transformed into cell mass and reduced the xylitol yield.

**Fig. 6:**
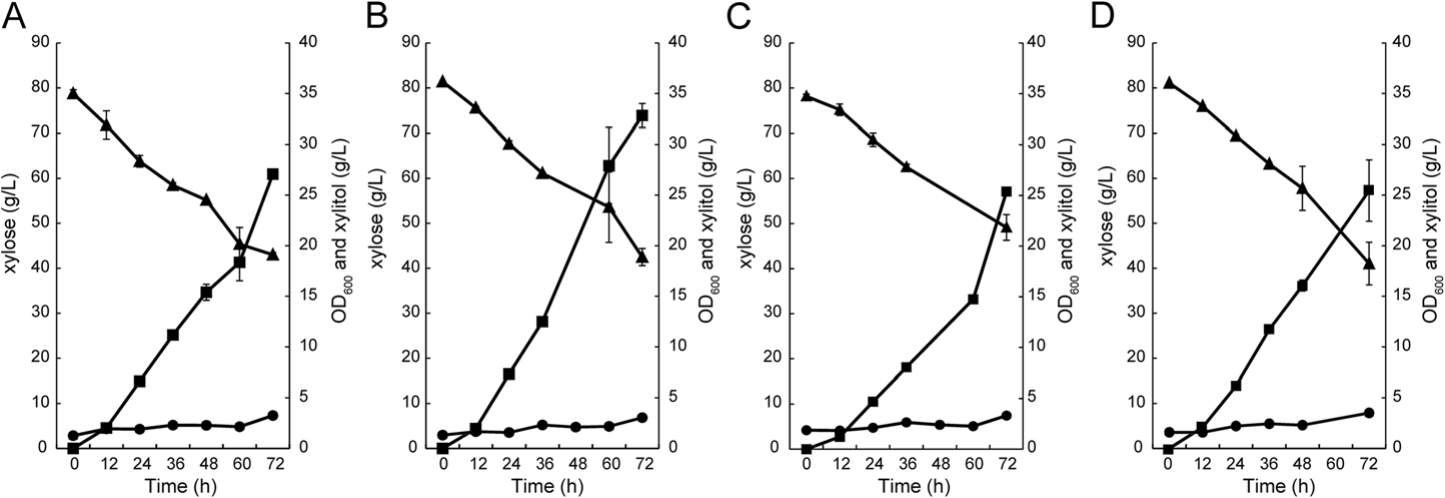
Inhibitor tolerance of *C. intermedia* FL023 in xylitol fermentation. *C. intermedia* FL023 was inoculated in xylose medium with **a** no inhibitor or 0.5 g L^−1^ **b** furfural, **c** acetate, and **d** syringaldehyde. Triangle, xylose; square, xylitol; and circle, cell density (OD_600_)

Xylose could be liberated from lignocellulosic materials by hemicellulase; however, pretreatment of lignocellulose, the first step for hydrolysis, generated several kinds of inhibitors. However, pretreatments generate inhibitors (phenolic compounds and formic acid in alkaline-pretreated biomass and hydroxymethyl furfural [HMF] and furfural in acid-pretreated biomass) that repress LA fermentation. Inhibitors present in the hydrolysate always repress microorganism growth and fermentation [41]. Thus, the tolerance of *C. intermedia* FL023 to phenolic inhibitors generated by alkaline pretreatment was tested. As shown in Fig. 6, *C. intermedia* FL023 grew and produced xylitol well from 80 g L^−1^ xylose medium containing 0.5 g L^−1^ of furfural, acetic acid, and syringaldehyde, compared to the control experiment using the fermentation medium containing no inhibitor. Surprisingly, the 1.21-fold higher xylitol yield was achieved by FL023 compared to the furfural-free control medium or acetic acid and syringaldehyde media (Fig. 6). It is probable that dehydrogenation of xylitol produces coenzyme NADH, which promotes the recycling of NAD^+^ and facilitates the reduction of furfural [38, 39]. Acetic acid and syringaldehyde showed little inhibitory effects on xylitol production by strain FL023. Taking the results together, *C. intermedia* FL402 showed strong tolerance to the tested inhibitors from pretreatment of lignocellulosic materials.

## Discussion

Lignocellulose is one of the most abundant renewable feedstocks that has attracted considerable attention as a substrate for biofuel and biochemical production. Lignocellulose contains mainly glucan and xylan that are embedded in a lignin-carbohydrate complex, which cannot be directly utilized by microorganisms. Therefore, three steps including pretreatment, enzymatic hydrolysis, hexose fermentation, and pentose fermentation are required for efficient utilization of lignocellulosic materials for SCP and xylitol production. In this study, we isolated a novel yeast strain *C. intermedia* FL023 which has a strong ability to utilize both pentose and hexose as well as cellulose, the most serious cellulase inhibitor, for cell growth, indicating this strain is a good candidate for SCP and xylitol, even ethanol production. Because of the high xylose and cellobiose utilization efficiency, the genomic DNA of strain FL023 has been electroporated into a thermotolerant *Saccharomyces cerevisiae* strain to generate a hybrid yeast strain with both good characteristics from the parental strains [31]. However, we found strain FL023 produced very little ethanol from xylose. Therefore, we focused on studying the SCP and xylitol production from lignocellulosic materials by this yeast strain. First of all, we found urea was the best nitrogen source for SCP production using the corncob hydrolysate as the carbon source through fed-batch separated hydrolysis and fermentation (SHF) process. Cell mass finally reached OD_600_ of 35 in the fed-batch SHF, which was equal to 34.6 g L^−1^. The productivity and yield were both higher than those previously reported in other yeasts. More importantly, strain FL023 produced 8.4 g L^−1^ dry SCP from *Miscanthus* straw using CSL as nitrogen source through SSF. *C. intermedia* FL023 provided a higher SCP yield from straw than ever reported, perhaps because (i) SSF was better than other methods for SCP production from lignocellulose substrate. During SSF, glucose and xylose were continuously released from glucan polymers and hemicellulose by Cellic2; the sugar released by hydrolysis is immediately consumed by the fermenting organisms, thus avoiding product inhibition. (ii) In SSF during the sugar releasing process, the low xylose concentration enhances xylose transport by the high-affinity transporters in *Candida* [32, 33].

*Candida* species are good candidates for high-titer and high-yield xylitol production [4]. For examples, *C. tropicalis* KCTC7221 and *Candida* sp. 559-9 produced 110 and 173 g L^−1^ xylitol from 150 and 200 g L^−1^ xylose, one of the main sugars liberated from lignocellulosic materials, respectively [11, 14]. However, other studies reported xylitol fermentations with low titer and yield. Therefore, the xylitol production ability of strain FL023 was tested. Finally, 45.7 g L^−1^ xylitol was produced by strain FL023 through fed-batch fermentation with the yield and productivity of 0.57 g g^−1^ xylose and 0.38 g L^−1^ h^−1^, respectively (Fig. 5d). In this study, it was found that xylitol production rate and productivity by strain FL023 increased with the increase of the initial xylose titer but the conversion efficiency was reduced (Fig. 5), due to that xylose reductase (XR) activity is significantly affected by xylose concentration [5, 37, 40]. Therefore, xylitol production through fed-batch fermentation with extended time and moderate concentration of xylose substrate was preferred. We also found that a high rate of xylitol production correlated with high cell density, while the xylitol production rate slowed down with decreasing xylose concentration (Fig. 5d). Moreover, xylitol is secreted by diffusion or passive transport using the concentration gradient across the membrane, which requires a higher intracellular xylitol concentration than that in the medium. In order to keep a higher titer of xylitol in the cells, a high concentration of xylose must be maintained during xylitol fermentation [13]. Therefore, continuous feeding and fermentation (CFF) could be employed to increase xylitol yield and productivity by *C. intermedia* FL023. At the first stage of the CFF process, the cell density should be increased at aerobic condition using glucose medium. Then, at the fermentation stage, xylose should be continuously supplied to maintain at 80 g L^−1^ and cell growth rate should be restricted by strictly controlling the air supply rate. At these conditions, the xylitol yield and productivity could be increased to satisfy industrial requirement.

Lignocellulose, the most abundant global source of biomass, could be used for biofuel and biochemical production. However, the crystalline structure of lignocellulosic biomass results in the major technical obstacle to biofuel and biochemical production. Therefore, pretreatments are required to break down the crystal structure. Pretreatments generate inhibitors (phenolic compounds and formic acid in alkaline-pretreated biomass and hydroxymethyl furfural [HMF] and furfural in acid-pretreated biomass) that repress LA fermentation. Thus, efficient LA production from pretreated biomass requires the removal of these inhibitors prior to fermentation or the use of inhibitor-tolerant bacteria. *C. intermedia* FL402 showed strong tolerance to furfural, acetic acid, and syringaldehyde, which represented the inhibitors from pretreated lignocellulosic materials by acids and alkalines (Fig. 6). This result indicated strain FL023 was feasible for SCP and xylitol production from lignocellulosic hydrolysates through fed-batch SHF or SSF, due to its high tolerance to the inhibitors in the hydrolysates.
